# SNP calling by sequencing pooled samples

**DOI:** 10.1186/1471-2105-13-239

**Published:** 2012-09-20

**Authors:** Emanuele Raineri, Luca Ferretti, Anna Esteve-Codina, Bruno Nevado, Simon Heath, Miguel Pérez-Enciso

**Affiliations:** 1 Centro Nacional de Análisis Genómico (CNAG), Parc Científic de Barcelona, Barcelona, 08028, Spain; 2 Centre for Research in Agricultural Genomics (CRAG) — Universitat Autònonoma de Barcelona, 08193 Bellaterra, Spain; 3 Institut Català de Recerca i Estudis Avançats (ICREA), Passeig Lluís Companys 23, 08010 Barcelona, Spain

## Abstract

**Background:**

Performing high throughput sequencing on samples pooled from different individuals is a strategy to characterize genetic variability at a small fraction of the cost required for individual sequencing. In certain circumstances some variability estimators have even lower variance than those obtained with individual sequencing. SNP calling and estimating the frequency of the minor allele from pooled samples, though, is a subtle exercise for at least three reasons. First, sequencing errors may have a much larger relevance than in individual SNP calling: while their impact in individual sequencing can be reduced by setting a restriction on a minimum number of reads per allele, this would have a strong and undesired effect in pools because it is unlikely that alleles at low frequency in the pool will be read many times. Second, the prior allele frequency for heterozygous sites in individuals is usually 0.5 (assuming one is not analyzing sequences coming from, *e.g.* cancer tissues), but this is not true in pools: in fact, under the standard neutral model, singletons (*i.e.* alleles of minimum frequency) are the most common class of variants because *P*(*f*) ∝ 1/*f *and they occur more often as the sample size increases. Third, an allele appearing only once in the reads from a pool does not necessarily correspond to a singleton in the set of individuals making up the pool, and vice versa, there can be more than one read – or, more likely, none – from a true singleton.

**Results:**

To improve upon existing theory and software packages, we have developed a Bayesian approach for minor allele frequency (MAF) computation and SNP calling in pools (and implemented it in a program called snape): the approach takes into account sequencing errors and allows users to choose different priors. We also set up a pipeline which can simulate the coalescence process giving rise to the SNPs, the pooling procedure and the sequencing. We used it to compare the performance of snape to that of other packages.

**Conclusions:**

We present a software which helps in calling SNPs in pooled samples: it has good power while retaining a low false discovery rate (FDR). The method also provides the posterior probability that a SNP is segregating and the full posterior distribution of *f* for every SNP. In order to test the behaviour of our software, we generated (through simulated coalescence) artificial genomes and computed the effect of a pooled sequencing protocol, followed by SNP calling. In this setting, snape has better power and False Discovery Rate (FDR) than the comparable packages samtools, PoPoolation, Varscan : for *N *= 50 chromosomes, snape has power ≈ 35*%*and FDR ≈ 2.5*%*. snape is available at http://code.google.com/p/snape-pooled/ (source code and precompiled binaries).

## Background

High throughput sequencing on samples pooled from different individuals is an efficient strategy to infer genetic variation in a population. In principle, single nucleotide polymorphisms (SNPs) and indels can be effectively detected and their frequency can be estimated with a variance not much higher than for individual sequencing [1-3]. However, interpretation of data from sequencing of pooled samples is quite different from that of individual samples and leads to additional issues. The most important difference is that in individual diploid samples, the frequency of alleles in each individual is known to be *f *= 0.5 for all SNPs, while in pools the frequency is a generic multiple of 1/*n*, where *n* is the number of different homologous chromosomes in the sample. For large samples, the frequency could be any number between 0 and 1. For this reason SNP calling methods for individual sequencing can detect only segregating sites with intermediate frequency and cannot be immediately extended to pooled samples without losing rare alleles. Rare alleles, though, are particularly important in population genetics because they represent most of the variability in natural populations. In fact, under the standard neutral model, singletons (*i.e.* alleles appearing only once among the reads) and rare alleles (say, with frequency *f *< 1/10) are the most frequent variants because *P*(*f*) ∝ 1/*f*. Moreover, the relative fraction of rare variants increases with read depth and sample size.

The detection of rare variants is also strongly affected by sequencing errors. While the impact of errors in individual sequencing can be reduced by setting a restriction on the minimum number of reads per allele, this would have a strong and undesired effect in pools because alleles at low frequency in the population often also appear at low frequency among the reads.

Furthermore, the process used to generate the reads from pooled samples has a critical effect, that is allele frequencies among the reads can be different from actual frequencies among individuals in the sample. If we assume that reads are randomly extracted from the sampled individuals, read counts will follow a binomial distribution with mean equal to the allele frequency in the sample times the read depth. This means that an allele appearing only once in the reads from a pool does not necessarily correspond to a singleton in the set of individuals making up the pool, and vice versa, there can be more than one read – or none – from a true singleton. Finally, since the frequency of each allele is unknown, SNP calling would depend on the prior for the frequency spectrum *P*(*f*) that is (explicitly or implicitly) specified. While maximum entropy (*i.e.* complete ignorance) would favor a flat prior, the standard population genetics result for neutral sites and low mutation rate is *P*(*f*) =* θ*/*f *[4] where *θ* is the genetic variability. Different choices could result in different calls in particular for low frequency SNPs. The need for a prior *P*(*f*) points to Bayesian methods to reconstruct the frequency spectrum. In this note we present a method for Bayesian estimation of posterior frequency distribution for SNPs in pooled samples. This method addresses the issues described above, and it can be naturally used to call SNPs from the posterior probability that alleles are actually segregating for each site.

### The model

Our model presents two interesting, novel aspects when it comes to pooled SNP calling: it takes into account sequencing errors, and allows the user to specify different priors. We explain below both these features, but first we introduce the basic formulae here. In what follows we will use *C* to indicate the read depth, *f * for the true alternative allele frequency, and *n*_*A*_ for the number of symbols alternative to the reference allele in the pileup. Moreover *N* is the number of different chromosomes in the pool and *θ *is the nucleotide diversity. Note that *N* obviously needs not coincide with the number of individuals in the pool: for example when considering autosomes in diploid populations, the number of chromosomes is twice the number of individuals. There could be also experimental setups where *N* is not known a priori, however in these situations *N* is typically large and therefore its exact value is not relevant for the model in this section. In general, if *N* is not precisely known, a rough estimate of it should suffice for our purposes. The quantity we want to compute is *P*(*f*∣*n*_*A*_,* θ*) that is, the posterior distribution of allele frequency. From there, many quantities of interest (*e.g.* mean, variance) can be obtained. Now, via Bayes one has

(1) $$P(f|n_{A},\theta )\propto P(n_{A}|f)P(f|\theta )$$

*P*(*n*_*A*_ ∣ *f*) can be written as follows:

(2) $$P(n_{A}|f)=\overset{N}{\underset{k=0}{\sum }}\left(\begin{array}{c} \;C\\ n_{A} \end{array}\;\right)p^{n_{A}}q^{C-n_{A}}\left(\begin{array}{c} N\\ \;k \end{array}\;\right)f^{k}{\left(1-f\right)}^{N-k}$$

where, in the simplifying assumption that there are no sequencing errors, $p\;=\;\frac{k}{N}$ and $q\;=\;1\;-\frac{k}{N}$ (this assumption is relaxed below). Equation (1) accounts for the fact that the observed number of alleles *n*_*A *_depends on the probability of including in the pool *k* alleles with frequency *f * in the population and on the probability of those alleles getting sequenced once they are in the pool (summed over all the possible values for *k*).

#### Warding off sequencing errors

A conspicuous feature of high throughput sequencing is the presence of a non-negligible error rate, hence we must allow for a difference between the nucleotide we observe as a result of the measurement and what is really present on the genome. To this purpose we introduce the notation *Ã* which means that we observe “A” as an output of the sequencing machine, whereas *A* means that the symbol is actually present on the genome in the same position. Here we have effectively only two symbols to account for, those which coincide with the reference genome (“R”) and those which belong to alternative alleles (“A”). To each letter generated by the sequencing machine there is associated a quality score , *i.e.* a character with ASCII code 33 to 126. This represents the probability that a particular base has been wrongly sequenced, and can be translated to numbers using the Phred scale as $\varepsilon =10^{-\frac{c-33}{10}}$ where *c* is the ASCII code. For each class of symbols we consider in our model (*i.e.* equal to the reference, or different from the reference) we compute the geometric mean of the respective error probabilities as they appear in the pileup, and we call it *ε*_*R *_(resp., *ε*_*A*_). Hence we translate the sequencing errors into probabilities as follows:

(3) $$\begin{array}{ll} P(A|Ã) & =1-\varepsilon_{A},P(R|Ã)=\varepsilon_{A}\;\\ P(R|\tilde{R}) & =1-\varepsilon_{R},P(A|\tilde{R})=\varepsilon_{R}\; \end{array}$$

Notice that once we set $P(A)=P(R)=\frac{1}{2}$ the quantities P(Ã∣A) (and cognates) obey the following relations, dictated once again by Bayes’ theorem:

(4) $$\begin{array}{lll} P(Ã|A) & =P(A|Ã)(P(Ã|A)+P(Ã|R))\; & \;\\ P(Ã|R) & =P(R|Ã)(P(Ã|A)+P(Ã|R))\; & \;\\ P(\tilde{R}|A) & =P(A|\tilde{R})(P(\tilde{R}|A)+P(\tilde{R}|R))\; & \;\\ P(\tilde{R}|R) & =P(R|\tilde{R})(P(\tilde{R}|A)+P(\tilde{R}|R))\; & \; \end{array}$$

from which we can infer P(Ã∣A)=1−ρ and P(Ã∣R)=* α *where $\rho =\frac{\varepsilon_{R}(1-2\varepsilon_{A})}{1-\varepsilon_{A}-\varepsilon_{R}}$ and $\alpha =\frac{\varepsilon_{A}(1-2\varepsilon_{R})}{1-\varepsilon_{A}-\varepsilon_{R}}$. Now, this allows us to rewrite *p* so that it allows for sequencing errors : we call this new probability $\tilde{p}$. We have

(5) $$\tilde{p}=P(Ã|A)p+P(Ã|R)q$$

We are aware of the fact that PHRED scores are useful only up to a point, and other kinds of errors can happen while sequencing which are not described by *ε*_*A*_,*ε*_*R*_. But many of these errors can be filtered out at an earlier stage. PCR duplicates are typically eliminated just after mapping; variants which are observed on one strand only (another sign of potential error) can be excluded from the pileup; the precision of the mapping can also be refined before using snape. We focus here on the problem of how to deal with the pooling because the error filtering is tackled by other packages which can easily be used together with snape.

#### Different priors

Having written *P*(*n*_*A *_∣* f*) taking into account sequencing errors, we now focus on the choice of prior for the allele frequencies, *P*(*f*∣*θ*). First, we discuss the distribution of the frequencies of segregating alleles *i.e.* those which satisfy the condition 0 <* f *< 1. If we assume complete ignorance about allele frequencies, a flat prior *P*(*f*∣*θ*) = 1 is a possible choice. However, it is well known that frequency spectra from real populations exhibit an excess of rare alleles, due to the fact that new mutations are born at low frequency and only a few reach intermediate frequencies before going to fixation or extinction. To account for this effect, we use some results from population genetics. The site frequency spectrum under the standard neutral model (*i.e.* a population evolving neutrally with constant population size) is equal to *θ*/*f*[5-8], where *θ* is the nucleotide variability in the population.

We will call this the informative prior with unfolded spectrum (*i.e.* knowing which is the ancestral and derived allele). An informative prior can also be written in the folded case *i.e.* when the identity of the ancestral allele is not known : in this case, it will have to be invariant with respect to the transformation *f *→ 1 −* f*. See Table 1 for the possible 4 combinations considered in our algorithm. The prior distribution of extreme frequencies (*f *= 0,1) is different since these frequencies correspond to fixed alleles in the population, *i.e.* to absorbing states of the dynamics of mutations and substitutions. The prior probability of *f *= 1 is naturally given by the genetic differentiation *D* between the outgroup sequence and the population studied, while the probability for *f *= 0 can be obtained by requiring the sum of all probabilities to be 1. Note that the continuum prior 1/*f* is improper since its integral diverges logarithmically; however we discretize the allele frequencies in the population as multiples of a minimum nonzero frequency 1/*N*_*d*_, so the discretized priors and posteriors that we use are well defined. *P*(*n*_*A *_∣* f*) is computed for *f * taking values in 0,0.01,…,0.99,1.

**Table 1:** **Priors for *f *= 0**,*** f*= 1, and 0 *< f <*1**

	**Informative**	**Flat**
unfolded	$\left\{\begin{array}{c} 1-\theta \beta -D\;\;\;f=0\\ D\;\;\;\;\;\;\;\;f=1\\ \frac{\theta }{100f}\;\;\;\;\;\;0<f<1 \end{array}\right)$	$\left\{\begin{array}{c} 1-\theta -D\;\;\;f=0\\ D\;\;\;\;\;\;f=1\\ \frac{\theta }{99}\;\;\;\;\;\;\;0<f<1 \end{array}\right)$
folded	$\left\{\begin{array}{c} \frac{\left(1-\theta \beta \right)}{2}\;\;\;\;\;\;\;f=0\\ \frac{\left(1-\theta \beta \right)}{2}\;\;\;\;\;\;\;f=1\\ \frac{\theta }{200f(1-f)}\;\;\;0<f<1 \end{array}\right)$	$\left\{\begin{array}{c} \frac{1-\theta }{2}\;\;\;f=0\\ \frac{1-\theta }{2}\;\;\;f=1\\ \frac{\theta }{99}\;\;\;\;\;\;\;0<f<1 \end{array}\right)$

We discretize the interval [0,1] with *N*_*d *_= 100 breakpoints. The numbers 99, 100 and 200 appearing in the formulæ in the table are normalization factors (respectively *N*_*d *_− 1, *N*_*d *_and 2*N*_*d*_). *β*is a normalization constant for the divergent function 1/*f*, $\beta =\overset{N_{d}}{\underset{i=1}{\sum }}\frac{1}{i}=\ln(N_{d})+\gamma$ where *γ *= 0.57721… is the Euler-Mascheroni constant.

## Methods

### Power and FDR

In order to test the performance of our method, we developed a pipeline. A 1 Mb sequence obtained by randomly sampling nucleotides for each position was used both as the ancestral sequence of the population simulated by ms[9] and as the reference genome on the alignment step (described below). The program ms was used to generate (through simulated coalescence) SNP data along a 1 Mb long DNA stretch for a single population with varying number of individuals (10, 25 and 50 diploid individuals) assuming nucleotide diversity 0.0005 and scaled recombination rate 0.0005 per site. For each resulting haplotype, the program ART[10] was used to generate simulated next generation sequencing (NGS) reads with the built-in profile for Illumina paired-end technology of 75 bp-long reads. To simulate the pooling process, reads were randomly selected from each sequence using either an equal proportion from each individual, or a skewed sampling scheme with some individuals over/under-sampled in the resulting pool. In the latter case, 50% of the individuals were sampled 50% more times whereas 50% of individuals were sampled 50% fewer times. An average depth of 20*X* was simulated for the whole pool in all cases, and reads were aligned with BWA [11]. We used the ancestral sequence as reference for the alignment, allowing for a maximum of 10 mismatches in each read, and removing resulting reads with mapping quality below 20. We also considered a more conservative mapping, up to 4 mismatches, but influence of this choice was small: we only observed a slight decrease in both power and false discovery rate (results not shown). Finally, SNPs were called with different methods restricting minimum and maximum depths to do the calling between 5*X* and 40*X*. We compared Varscan[12], PoPoolation[13], samtools pileup[14], samtools mpileup[14], and snape with flat and informative priors. In Varscan, behaviour depended largely on the significance level used : at low P-values power was very low, whereas at P-value 1, FDR was very high. Here we chose a P-value of 0.1 as a compromise. In samtools pileup we retained SNPs with quality >20, and samtools mpileup was run with default options. snape was run with flat and informative priors with options divergence 0.01, prior nucleotide diversity 0.001 and folded spectrum. We retained SNPs with posterior probability of segregation > 0.9 (to reproduce these results, see also the manual in the software repository). Power was computed as the proportion of true SNPs in the population (*i.e.*, before pooling) located within regions of appropriate depth that were correctly recovered. False Discovery Rate (FDR) was obtained as the proportion of SNP calls that were incorrect. A total of 100 replicates per case were simulated, and average power and FDR were plotted. Besides, we also plotted power and FDR as functions of actual depth per site, and of minor allele frequency (MAF).

### Frequency spectrum

Given that the SNP calling process produces a bias against alleles found at low frequencies, we wished to study its effect on the site frequency spectrum (SFS). This is important because most real SNPs will be singletons or low frequency sites. We performed 100 coalescent simulations using the same settings as above with 100 chromosomes. For each simulation we subsampled, with replacement, 20 chromosomes in order to mimic the 20*X* read depth used in the simulations described above, and plotted the resulting folded SFS excluding non-polymorphic sites. To compare the performance of snape and samtools in recovering the SFS, we estimated the SFS obtained by snape and samtools for the same set of simulations. For each software, the SFS was calculated using the SNPs identified by each software that were covered by exactly 20 reads, and taking as estimate for the frequency of each SNP the raw frequency of reads carrying the alternative allele. We restricted the estimate at depth 20*X* simply for estimates of SFS to be comparable. Note that the interpretation of SFS is much more complicated if a mixture of depths is analyzed, *e.g.*, for depth 4*X *the folded frequency can only be 0.25 and 0.50, whereas the range is smoother at higher depths. While the approach of using raw read frequency as the estimate of allele frequency is not optimal, it allows us to compare easily samtools and snape. Note that, in contrast to snape, samtools does not output the posterior distribution of allele frequency f when using pooled samples.

## Results and discussion

### Power and FDR

**Figure 1:**
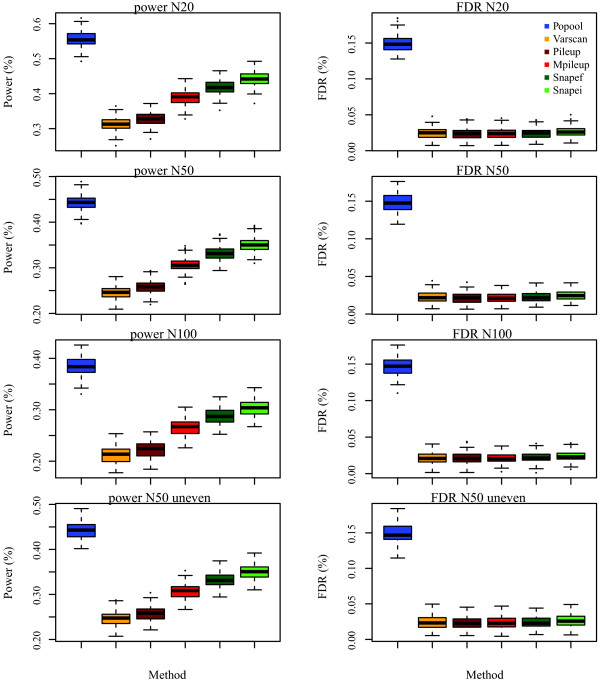
**Average power (left) and false discovery rate (right) for each of the methods considered.** Results are shown when using *N *= 20,50 and 100 chromosomes (from top to bottom) for average sequencing depth 20*X*. Bottom row shows the result of unequal contribution of individuals to a pool of 50 chromosomes. With uneven contribution, half of the individuals were sampled 50% more times and the remaining half were sampled 50% fewer times. Average of 100 replicates.

Average power and FDR are shown in Figure 1.

PoPoolation exhibits the highest power overall, however this comes at the cost of a very high FDR, a behaviour which is not unexpected as this software was not conceived as a SNP caller (R. Köfler, personal comm.). Other than PoPoolation, snape exhibited the largest power, whereas the samtools mpileup function turned out to perform much better than the deprecated pileup function. Adding the prior information on the site frequency spectrum improved power by ≈ 15*%* in snape while not affecting FDR. This increase in power was observed across the range of parameters considered.

Note that power decreases with *N*, the number of chromosomes in the pool. This occurs because the number of SNPs increases as well with *N* (including singletons, which are difficult to detect), and the increase is proportional to Ewen’s constant $\overset{N-1}{\underset{i=1}{\sum }}\frac{1}{i}$. After taking into account this fact, the number of SNPs called is actually the same for a given depth, irrespective of the number of individuals in the pool.

**Figure 2:**
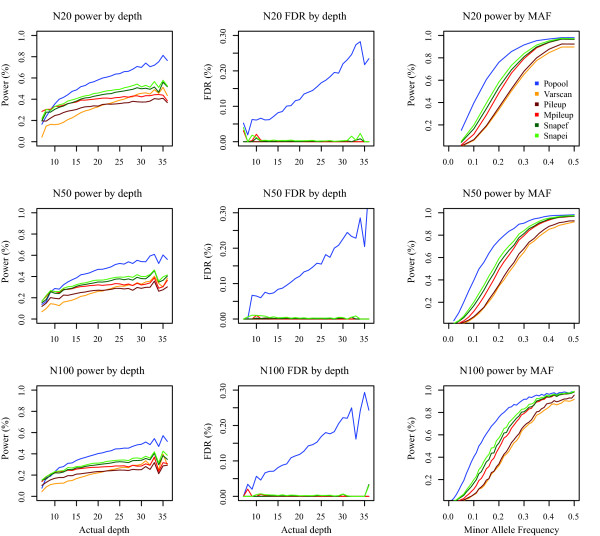
**Power and false discovery rate (FDR) according to actual depth and minimum allele frequency when using* N *= 20,50 and 100 chromosomes (from top to bottom) obtained with different methods (legend on upper-right panel).** Average depth was 20*X*. Left column panels show power as a function of actual depth, middle column is the false discovery rate as a function of actual depth, and right column, power as a function of true minor allele frequency (MAF). Average of 100 replicates.

Two main factors affect the accuracy of SNP calling in pools: depth and minimum allele frequency, although their effect varied according to the algorithm used (Figure 2).

PoPoolation’s FDR increased dramatically with depth because, as mentioned, it is not conceived as a SNP caller and does not correct properly for sequencing errors. For the rest of the algorithms, FDR did not depend on depth. In contrast, power increased with depth, although it reached a plateau after 30*X *approximately except in PoPoolation and Varscan.

For what regards the influence of allele frequencies, it is precisely at low frequency that snape performs better than other SNP callers (when keeping in account both power and FDR). Note that power of different methods tend to converge at intermediate frequencies, simply because an intermediate allele frequency is equivalent to that in a diploid heterozygous individual, and SNP callers usually assume diploid individuals. For instance, for* N *= 20,0.96 and 0.95 of SNPs for MAF > 0.40 are correctly called with snape and mpileup, respectively. For SNPs with MAF < 0.15, those numbers become 0.22 and 0.14 respectively.

Two effects are worth mentioning : the reduction in power consequent to pooling, and what happens if samples are represented unevenly in the pool. For what regards the first, simple theoretical analyses show that the site frequency spectrum is highly distorted for moderate or low depths (< 20), even if SNP calling were perfect. Pérez-Enciso and Ferretti [15] also show that, even at very high depths, some chromosomes will not be sampled when the number of individuals in the pool is large, causing the loss of singletons and of low frequency alleles.

When it comes to the second concern, it has to be noted that it is difficult to develop methods that account for uneven sampling in pools without knowing the actual distribution of individual contributions. Besides, there is no direct way to detect it from the data without extra assumptions about the demographic / evolutionary pattern of the data (for example, assuming a single neutral population without admixture). Nevertheless, the really important question is: how strong is the impact of uneven sampling on the method? SNP calling should not be strongly affected because unequal sampling of chromosomes often results in a shift towards intermediate frequencies. Consider, *e.g.*, the extreme case of a pool containing only contributions from 2-3 chromosomes: while rare alleles remain rare, frequent SNPs will be detected even at higher power than in a balanced pool. On the other hand, the mean allele frequency is the same as for balanced pools, although the variance in the estimation of population allele frequencies will increase. This increase in variance cannot be captured by any available method. Figure 1 (bottom row) shows the simulation results of an unbalanced pool (*N *= 50). The figure confirms previous arguments whereby average power and FDR remain approximately constant, whereas variance increases slightly, as can be seen from the wider distribution of power values than that of balanced pooling.

### Frequency spectrum

**Figure 3:**
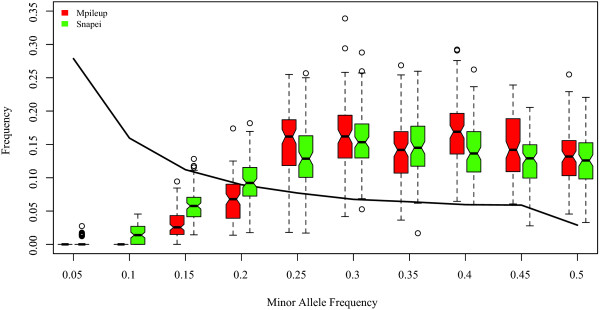
**Effect of pooling and SNP calling on estimated site frequency spectrum (SFS).** The thick black line depicts the true SFS after pooling, but before sequencing, *i.e.*, as if power was 1 and no false discoveries. Boxplots show the estimated SFS after sequencing and SNP calling for reads with exact depth 20. The two best methods are compared: samtools mpileup and snape with informative prior. Results for 100 replicates and *N *= 100 chromosomes.

The frequency spectra of SNPs called with informative snape and mpileup approaches are shown in Figure 3.

The true spectrum is the black line. As expected from previous figures, which show a reduced power at low frequency alleles, singletons and low frequency alleles are clearly under represented with both methods. Yet, snape obtains a less biased spectrum than other methods because a higher percentage of rare allele SNPs is called. The bias towards high MAF sites decreased if we lowered the posterior probability threshold for a SNP to be called , *P *= 0.50 instead of *P *= 0.90, but at the price of increasing FDR (results not shown). All in all, missing low MAF SNPs is inherent to all methods but the approach proposed here performs better than standard tools. Also, snape computes the complete posterior probability of the allele frequency *f *, which is of interest to develop new statistics that consider the whole uncertainty on *f *, rather than point estimates.

## Implementation

We developed a software called snape-pooled that reads a file in pileup ( http://samtools.sourceforge.net/pileup.shtml) format as input and a number of command line options specifying the total number of chromosomes being sequenced, *θ* (the prior nucleotide diversity, default 0.001), *D* (the prior divergence between the sample sequenced and the reference, default 0.1), the prior type (which can be flat or informative) and whether the reference allele can be taken as ancestral one or not (*i.e.*, unfolded or folded SFS). The output file contains *P*(*f*∣*n*_*A*_), together with other useful indicators (see the manual).

## Conclusions

Using standard tools for calling SNPs in pools is not appropriate because many true variants will be confounded with sequencing errors and therefore will not be called. Here, we present a Bayesian method that overcomes this difficulty while retaining a low FDR. It also provides the posterior probability that a SNP is segregating and the full binned posterior distribution of *f * for every SNP. This should allow us to develop more reliable tests based on site frequency spectrum compared to those that merely employ point estimates.

## Availability

The software (source code included) is available at http://code.google.com/p/snape-pooled/.

## Competing interests

The authors declare that they have no competing interests.

## Authors’ contributions

ER, LF, MP developed the mathematical method. ER wrote the software. BN wrote the simulation pipeline. AE and BN tested the software and ran the comparison against other packages. SH took part in discussions and testing. All authors read and approved the final manuscript.

## References

[B1] Ferretti L Raineri E Ramos-Onsins S Neutrality tests for sequences with missing data Genetics 2012 191 4 1397 1401 10.1534/genetics.112.139949 22661328 PMC3416018

[B2] Amaral AJ Ferretti L Megens H Crooijmans R Nie H Ramos-Onsins S Perez-Enciso M Schook L Groenen M Genome-wide footprints of pig domestication and selection revealed through massive parallel sequencing of pooled DNA PLoS ONE 2011, 6 10.1371/journal.pone.0014782 PMC3070695 21483733

[B3] Futschik A Schlötterer C The next generation of molecular markers from massively parallel sequencing of pooled DNA samples Genetics 2010 186 207 218 10.1534/genetics.110.114397 20457880 PMC2940288

[B4] Crow J Kimura M An introduction to population genetics theory 1970 Harper & Row, Publishers, New York, Evanston and London

[B5] Fisher R The distribution of gene ratios for rare mutations Proc R Soc Edinb 1930 50 205 220

[B6] Wright S The distribution of gene frequencies under irreversible mutation Proc Natl Acad Sci USA 1938 24 7 253 10.1073/pnas.24.7.253 16577841 PMC1077089

[B7] Kimura M Diffusion models in population genetics J Appl Probability 1964 1 2 177 232 10.2307/3211856

[B8] Kimura M The number of heterozygous nucleotide sites maintained in a finite population due to steady flux of mutations Genetics 1969 61 4 893 5364968 10.1093/genetics/61.4.893 PMC1212250

[B9] Hudson RR Generating samples under a Wright-Fisher neutral model Bioinformatics 2002 8 2 337 8 11847089 10.1093/bioinformatics/18.2.337

[B10] Huang W Li L Jason R Marth GT ART: a next-generation sequencing read simulator Bioinformatics 2011 28 4 593 4 22199392 10.1093/bioinformatics/btr708 PMC3278762

[B11] Li H Durbin R Fast and accurate short read alignment with Burrows-Wheeler Transform Bioinformatics 2009 26 5 589 95 20080505 10.1093/bioinformatics/btp698 PMC2828108

[B12] Koboldt DC Chen K Wylie T Larson D McLellan M Mardis E Weinstock G Wilson R Ding L VarScan: variant detection in massively parallel sequencing of individual and pooled samples Bioinformatics 2009 25 2283 2285 10.1093/bioinformatics/btp373 19542151 PMC2734323

[B13] Kofler R Orozco-Terwengel P De Maio N Vinay Pandey R Nolte V Futschik A Kosiol C Schlötterer C PoPoolation: a toolbox for population genetic analysis of next generation sequencing data from pooled individuals Plos One 2011 1 6 e15925 21253599 10.1371/journal.pone.0015925 PMC3017084

[B14] Li H Handsaker B Wysoker A Fennell T Ruan J Homer N Marth G Abecasis G Durbin R Subgroup GPDP The Sequence Alignment/Map (SAM) format and SAMtools Bioinformatics 2009 25 16 2078 9 10.1093/bioinformatics/btp352 19505943 PMC2723002

[B15] Ferretti L Pérez-Enciso M Massive parallel sequencing in animal genetics: wherefroms and wheretos Anim Genet 2010 41 6 561 9 10.1111/j.1365-2052.2010.02057.x 20477787

